# Excitation energy transfer in proteoliposomes reconstituted with LH2 and RC-LH1 complexes from *Rhodobacter sphaeroides*

**DOI:** 10.1042/BSR20231302

**Published:** 2024-02-19

**Authors:** Xia Huang, Cvetelin Vasilev, David J.K. Swainsbury, C. Neil Hunter

**Affiliations:** 1 Department of Biological Sciences, Xi’an Jiaotong-Liverpool University, Suzhou, Jiangsu 215123, China; 2 Jinan Guoke Medical Technology Development Co., Ltd, Jinan, Shandong 250101, China; 3 School of Biosciences, University of Sheffield, Sheffield S10 2TN, U.K.; 4 School of Biological Sciences, University of East Anglia, Norwich, NR4 7TJ, U.K.

**Keywords:** LH2, Light harvesting, photosynthesis, RC-LH1, Rhodobacter sphaeroides

## Abstract

Light-harvesting 2 (LH2) and reaction-centre light-harvesting 1 (RC-LH1) complexes purified from the photosynthetic bacterium *Rhodobacter (Rba.) sphaeroides* were reconstituted into proteoliposomes either separately, or together at three different LH2:RC-LH1 ratios, for excitation energy transfer studies. Atomic force microscopy (AFM) was used to investigate the distribution and association of the complexes within the proteoliposome membranes. Absorption and fluorescence emission spectra were similar for LH2 complexes in detergent and liposomes, indicating that reconstitution retains the structural and optical properties of the LH2 complexes. Analysis of fluorescence emission shows that when LH2 forms an extensive series of contacts with other such complexes, fluorescence is quenched by 52.6 ± 1.4%. In mixed proteoliposomes, specific excitation of carotenoids in LH2 donor complexes resulted in emission of fluorescence from acceptor RC-LH1 complexes engineered to assemble with no carotenoids. Extents of energy transfer were measured by fluorescence lifetime microscopy; the 0.72 ± 0.08 ns lifetime in LH2-only membranes decreases to 0.43 ± 0.04 ns with a ratio of 2:1 LH2 to RC-LH1, and to 0.35 ± 0.05 ns for a 1:1 ratio, corresponding to energy transfer efficiencies of 40 ± 14% and 51 ± 18%, respectively. No further improvement is seen with a 0.5:1 LH2 to RC-LH1 ratio. Thus, LH2 and RC-LH1 complexes perform their light harvesting and energy transfer roles when reconstituted into proteoliposomes, providing a way to integrate native, non-native, engineered and *de novo* designed light-harvesting complexes into functional photosynthetic systems.

## Introduction

Photosynthesis is the process used by phototrophic organisms to capture solar energy and convert it into chemical energy, which ultimately supports almost all known life on Earth. The photosynthetic process begins with the absorption of light by light-harvesting (LH) complexes followed by the migration of excitation energy through a network of antenna complexes towards the reaction centre (RC) where trapping occurs by initiating a photochemical charge separation [1]. In many purple photosynthetic bacteria, including the model organism *Rhodobacter* (*Rba*.) *sphaeroides*, four complexes are required for harvesting, trapping and storing photosynthetic energy. These are the peripheral light-harvesting 2 complex (LH2), the RC-light-harvesting 1 (RC-LH1) core complex, the cytochrome *bc*_1_ complex, and ATP synthase [1–4]. In *Rba. sphaeroides* these complexes are housed within ∼50 nm diameter intracytoplasmic membrane (ICM) vesicles, often called chromatophores [5]. Within the chromatophore arrays of LH2 and RC-LH1 complexes are in direct contact [5] to minimize inter-pigment distances, creating a network for efficient transfer and trapping of excitation energy, whilst the cytochrome *bc*_1_ complex resides in lipid-rich domains [2,3,6,7] adjacent to the RC-LH1 complexes [8].

The 3D structures of the LH2 complex and both the monomeric and dimeric forms of the RC-LH1 complex of *Rba. sphaeroides* have been determined [9–14]. The peripheral LH2 antenna comprises nine heterodimers of single transmembrane spanning α and β polypeptides, with each heterodimer binding one bacteriochlorophyll (BChl) dimer absorbing at 850 nm (B850), a monomeric BChl absorbing at 800 nm (B800) and one carotenoid absorbing between 400 and 600 nm. The monomeric LH1 antenna consists of an open ring of 14 αβ heterodimers, each binding two carotenoids and a dimer of BChls absorbing at 875 nm (B875). The 14 αβ heterodimers do not completely surround the RC, which leaves a gap to allow the export of quinols and import of quinones, facilitated by the PufX and protein-Y polypeptides [10]. These monomeric RC-LH1 complexes can associate via interactions mediated, in part, by the ring-interrupting PufX polypeptide to form a 28-subunit S-shaped antenna surrounding two RCs [11]. This dimeric structure forms the majority of core complexes in photosynthetic membranes of *Rba. sphaeroides* [5,15–17]; excitation sharing between the two halves of the dimer is an efficient mechanism for coping with high levels of excitation [18,19].

Light-harvesting in the chromatophore is mediated by bacteriochlorophylls (BChls) and carotenoid pigments in LH2 antenna complexes followed by excitation energy transfer via the LH1 antenna complexes to the RC, along the energy migration route B800 (LH2) → B850 (LH2) → B875 (LH1) → RC [20]. The carotenoids act as accessory antenna pigments, absorbing light in the 400–600 nm range, where BChls do not absorb, passing excitation energy to the BChls [21]. Once this energy reaches the RC from the antenna complexes charge separation is initiated, ultimately trapping the energy in the form a reduced quinone molecule [22]. The timescale for energy migration and trapping is rapid, taking approximately 60 ps from initial absorption by LH2 to trapping at the RC [23]. Within this time energy may migrate tens of nanometres via several LH2 complexes with high energy transfer efficiencies [20]. The other important function of carotenoids is to quench dangerous excited states to protect the photosynthetic organism from photo-oxidative damage [21,24,25].

Whilst the structures of complexes and decades of spectroscopic investigation provide an established model for energy and electron transfers, a full understanding of how antenna complexes interact to transfer energy over tens of nanometres requires that we know their spatial arrangement within photosynthetic membranes. To this end many studies have utilized atomic force microscopy (AFM) to observe the supramolecular organization of individual complexes in bacterial photosynthetic membranes, for example [26–28]. Membranes from mutant [29–32] and wild-type [5,15,16] strains of *Rba. sphaeroides* were analysed using this technique; AFM topographs combined with high-resolution structures of complexes and quantitation by mass spectrometry led to the construction of *in silico* models of the ICM vesicle [2,3,33,34], culminating in a 100-million atom simulation of the entire chromatophore [7]. Such models can predict energy transfer and trapping behaviour and identify desirable design motifs for artificial photosynthetic systems. One way to augment the study of natural systems is to assemble arrays of complexes on planar gold, glass or silicon substrates. Linear assemblies of single types of antenna complex, for example, the LH2 complex of *Rba. sphaeroides*, or the LHCII complex of plants, retain their functionality on solid substrates in terms of absorption, emission and energy transfer [35–40].

Developing methods to control the organisation and stoichiometry of two different complexes deposited on a surface, such as LH2 and RC-LH1 complexes, would aid our understanding of the mechanisms of excitation energy transfer and trapping and provide molecular-level strategies for the development of novel photosynthetic systems. In native systems, LH2:RC-LH1 ratios alter as an adaptation to fluctuating light intensities [16,41], but in addition to providing another way to study LH2:RC-LH1 ratios reconstituted planar arrays offer the additional option of bringing together complexes from different organisms. For example, Uragami and co-workers showed that it is possible to combine LH2 complexes (from *Rhodopseudomonas acidophila*) and LH1-RC complexes (from *Blastochloris viridis*) within a lipid bilayer system [42]. Recently, we investigated the excitation energy transfer between LH2 and RC-LH1 complexes patterned on the micron scale onto a glass substrate [43], showing that EET processes can take place within an artificial photosynthetic network.

Here we have reconstituted complexes from *Rba. sphaeroides* into liposomes, while exerting control over the LH2: RC-LH1 stoichiometry. AFM topographs of proteoliposomes show the self-assembly of photosynthetic units with LH2 and RC-LH1 complexes in close proximity. By employing donor LH2 complexes containing the carotenoid spheroidenone and acceptor RC-LH1 complexes lacking carotenoids [44], we were able to selectively excite LH2 and observe fluorescence emission from RC-LH1 complexes, demonstrating energy transfer from LH2 to RC-LH1 complexes.

## Results

### Production of RC-LH1 and LH2 with specific carotenoids

**Figure 1:**
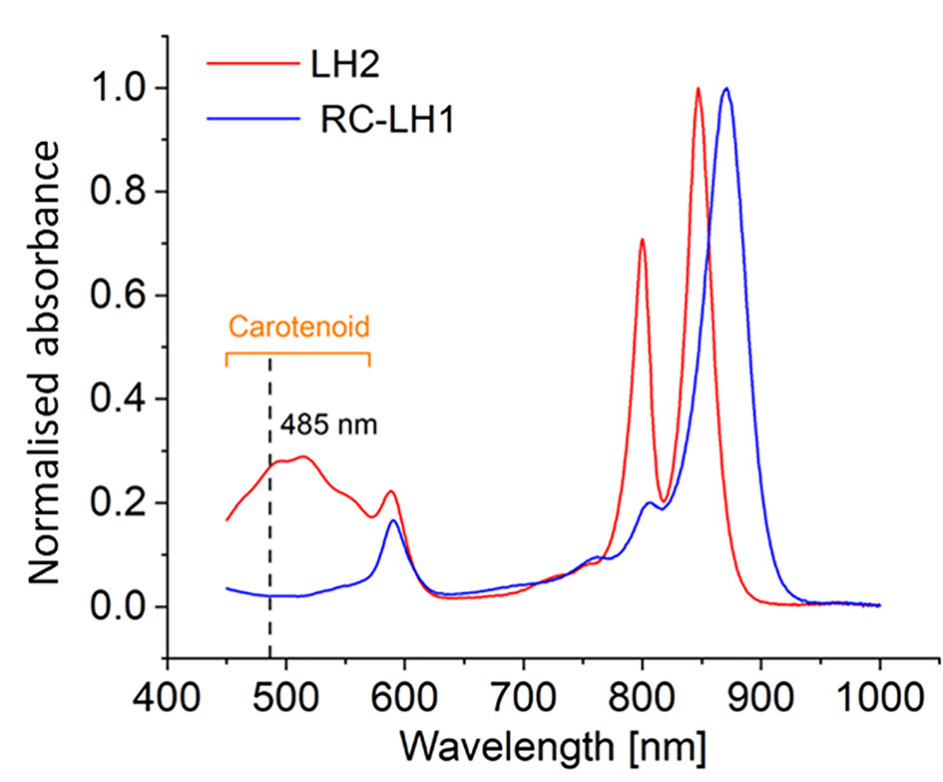
Absorption spectra of purified LH2 and RC-LH1 complexes The spectra were normalized at the near-IR maxima at 850 nm for LH2 (red) and 870 nm for RC-LH1 (blue).

Monitoring energy transfer from LH2 to RC-LH1 in a mixed array of complexes requires an excitation wavelength that specifically excites LH2, but this is difficult to achieve because of overlapping absorption bands. To overcome this limitation, we prepared ‘red’ LH2 complexes from semi-aerobically grown cells of wild-type *Rba. sphaeroides*, in which the main carotenoid, spheroidenone, absorbs in the 400–600 nm range between the Soret and Q_x_ transitions of the BChls [44] (Figure 1). The RC-LH1 complexes were prepared from a strain harbouring a markerless deletion of phytoene desaturase (*crtB*), which abolishes the production of coloured carotenoids, yielding ‘blue’ RC-LH1 complexes with negligible absorption in the 400–600 nm range [44] (Figure 1). At the excitation wavelength of 485 nm the spheroidenone-containing LH2 complexes absorb at 27% of the B850 maximum whereas the RC-LH1 complexes absorb at only 2.1% of their B875 maximum, permitting the preferential excitation of LH2 and greatly simplifying experimental design and data analysis.

### Reconstitution of RC-LH1 and LH2 in proteoliposomes

**Figure 2:**
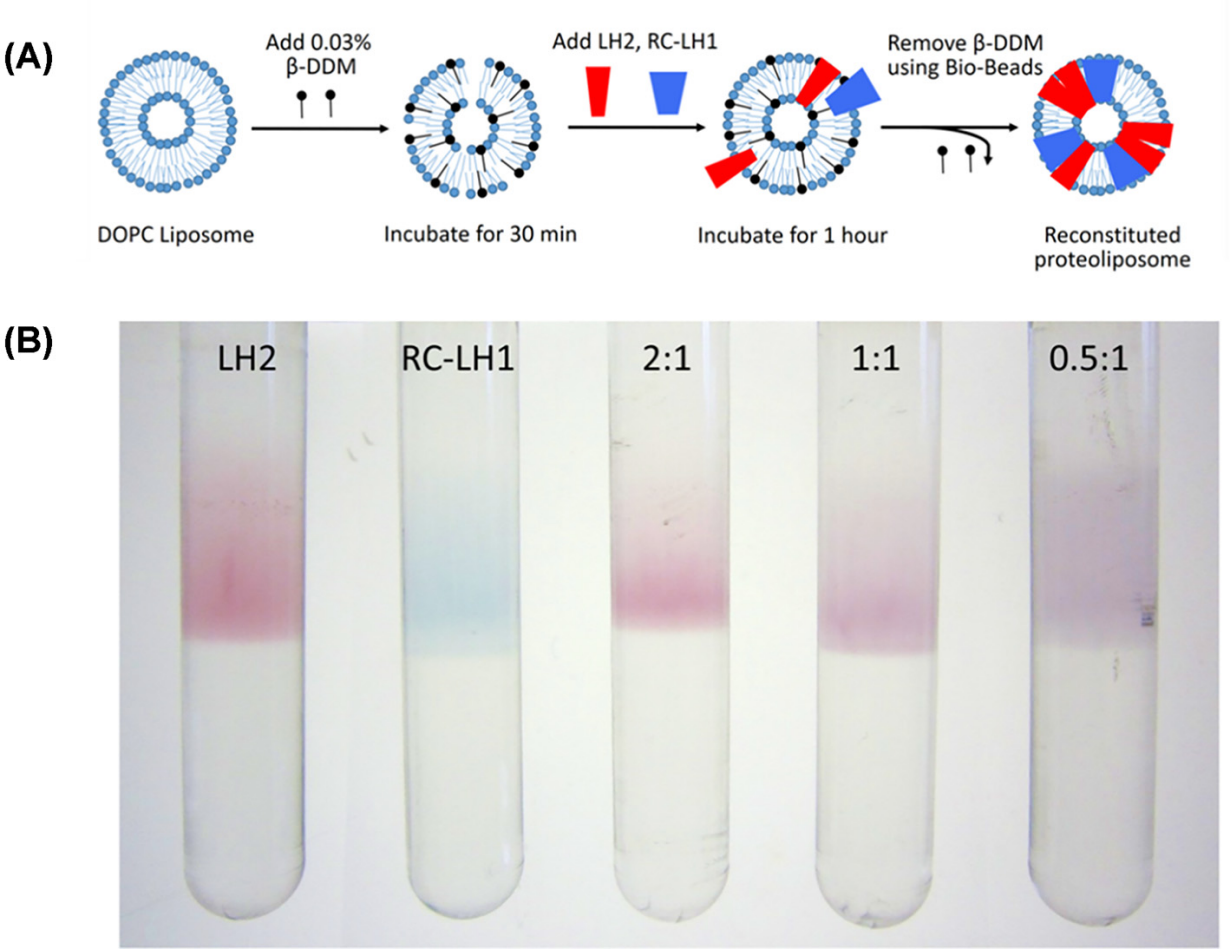
Reconstitution of the LH2 and RC-LH1 complexes into liposomes (**A**) Schematic diagram showing reconstitution of LH2 and RC-LH1 complexes into proteoliposomes. (**B**) Sucrose gradient fractionation of LH2 or RC-LH1 only proteoliposomes and proteoliposomes with 2:1, 1:1 and 0.5:1 molar ratios of LH2:RC-LH1.

We formed liposomes from the lipid 1,2-dioleoyl-*sn*-glycero-3-phosphocholine (DOPC) by extrusion through a size-selective membrane with a 200 nm pore size. The purified RC-LH1 and LH2 complexes were then reconstituted into the liposomes by the slow removal of detergent, promoting their spontaneous insertion into the bilayer of the liposome, followed by fractionation of the mixture using sucrose density gradients (see Methods and Figure 2). To optimize conditions for proteoliposome assembly we first performed RC-LH1-only or LH2-only reconstitutions (Figure 2B). Using a lipid-to-protein molar ratio of 500:1, we observed a single pigmented band at the 25–30% sucrose interface corrosponding to correctly formed proteoliposomes, with little to no free complexes at lower sucrose concentrations or aggregated complexes pelleting at the bottom of the tube. This demonstrated that our procedure allows the efficient and controlled transfer of complexes from detergent micelles to the lipid bilayer. Using these optimised conditions we next tested whether we could control the ratio of LH2 and RC-LH1 simply by adding the desired quantity of each to the reconstitution. Therefore, we prepared proteoliposomes with 2:1, 1:1 and 0.5:1 molar ratios of LH2:RC-LH1, shown in Figure 2B. To ensure the proteoliposomes conformed to the expected size, the LH2 reconstituted proteoliposomes were measured by dynamic light scattering (DLS). The distribution of liposome sizes showed that they became somewhat larger following fractionation on sucrose gradients (Supplementary Figure S1 and Table S1), possibly due to the removal of protein-free liposomes. The DLS profiles, and AFM topographs in Figure 4, are consistent with the proteoliposomes forming closed, spherical vesicles as illustrated schematically in Figure 2A.

### Absorption spectra of the proteoliposomes

**Figure 3:**
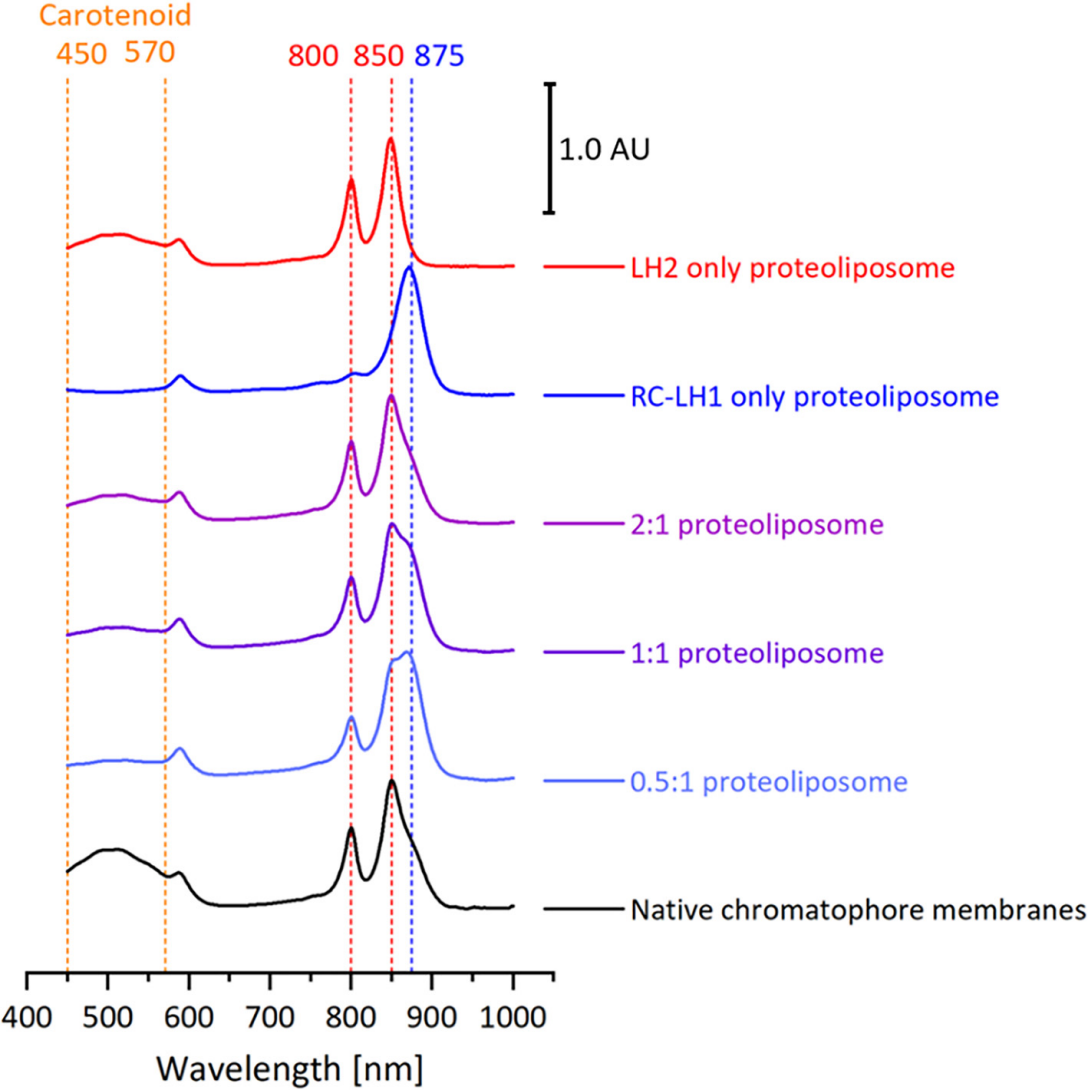
Normalized absorption spectra of native *Rba. sphaeroides* membranes, purified LH2 and RC-LH1 complexes, and reconstituted proteoliposome samples The spectra were normalized at the near-IR maxima and have been offset for clarity. LH2:RC-LH1 ratios are shown for reconstituted proteoliposomes.

Figure 3 shows UV/Vis/NIR absorption spectra of the proteoliposomes. Preparations containing only RC-LH1 or LH2 complexes yield spectra almost identical to the same complexes in detergent, but with a 2 nm red-shift for the LH2 B850 band. This analysis shows that the integrity of the complexes was retained upon insertion into the lipid bilayer. The small spectral shift is attributed to the transfer from a detergent to a lipid environment, as previously observed by Pflock and co-workers [45]. When liposomes containing both complexes are produced the resultant spectra clearly contain peaks corresponding to both LH2 and RC-LH1 with the relative 850 and 875 nm absorption varying with the ratio of complexes in the reconstitution mixtures. We deconvoluted the proteoliposome spectra by fitting reference spectra of the individual complexes and a model scatter curve to estimate levels of LH2 and RC-LH1 complexes. Table 1 shows that LH2: RC-LH1 ratios within the proteoliposomes reflected the relative amounts of complexes used in reconstitutions, so this method provides a good level of control over the composition of liposomes. We also compared our proteoliposomes with chromatophore membranes prepared from semi-aerobic grown wild-type *Rba. sphaeroides* cells. We estimate that these membranes contain a 2.13:1 LH2:RC-LH1 ratio by deconvolution, in good agreement with other studies of chromatophore membranes prepared under the same conditions [16,41]. These data clearly demonstrate the assembly of proteoliposomes that spectrally resemble native membranes.

**Table 1:** LH2:RC-LH1 ratios in reconstituted proteoliposomes calculated from the absorption spectrum deconvolution results and compared with chromatophore membranes from semi-aerobic grown wild-type *Rba. sphaeroides* cells

Sample	LH2/RC-LH1 ratio	
	Spectral deconvolution result	Protein ratio used during incubation
LH2-only	N/A	LH2-only
RC-LH1 only	N/A	RC-LH1 only
Reconstitution	1.92 : 1	2 : 1
Reconstitution	0.90 : 1	1 : 1
Reconstitution	0.49:1	0.5:1
Chromatophores	2.13:1	--

### Arrangement of individual complexes in the proteoliposomes

**Figure 4:**
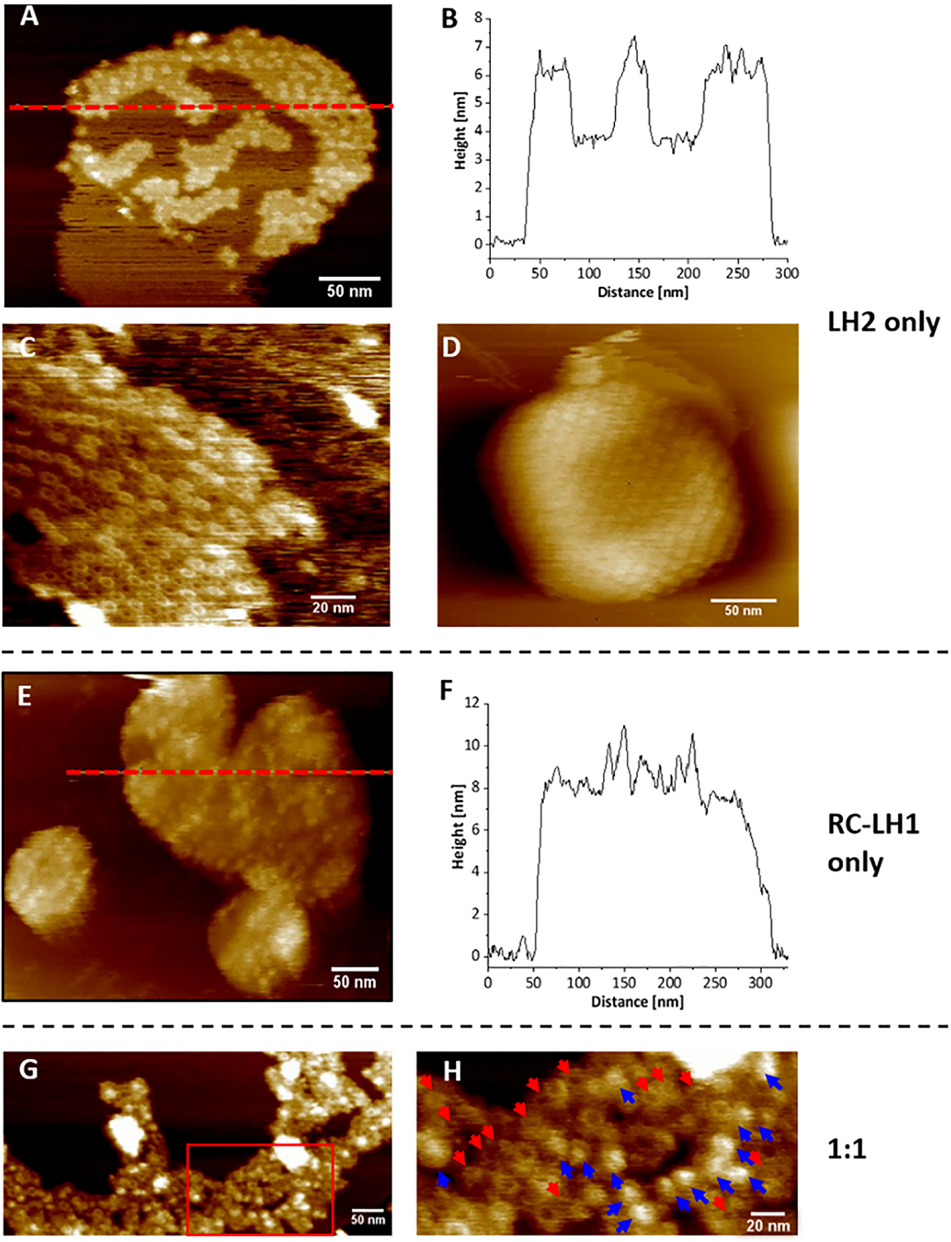
AFM topographs of proteoliposomes (**A**) LH2-only proteoliposome patch revealing three distinct height levels – the mica substrate (dark), the DOPC bilayer (brown) and the assemblies of LH2 complexes (pale brown). (**B**) The corresponding height profile along the red dashed line in panel A, showing three 6–7 nm height maxima corresponding to LH2, and two 3.5–4 nm heights corresponding to lipid-only regions. (**C**) A cluster of LH2 in a DOPC bilayer with two height levels of LH2 apparent. (**D**) A liposome with arrays of LH2 complexes arranged in a rectangular pattern. (**E**) The surface morphology of RC-LH1 only proteoliposomes. (**F**) The corresponding height profile along the red dashed line in (E). (**G**) Topograph of a 1:1 LH2:RC-LH1 proteoliposome patch. (**H**) The zoomed image of the area marked with a red rectangle in (G). Blue arrows indicate RC-LH1 complexes and red arrows indicate LH2. Figures of additional samples for each ratio are included in Supplementary Figure S3.

We used AFM to visualise the arrangement of the individual complexes within the proteoliposomes. Figure 4A–D shows AFM topographs and a height profile of LH2-only proteoliposomes. The topograph in Figure 4A clearly reveals three distinct height levels – the mica substrate (dark), the DOPC bilayer (brown) and the assemblies of LH2 complexes (pale brown), showing that the liposome is clearly segregated into LH2-rich and lipid-rich domains. Figure 4B shows the corresponding height profile along the red dashed line in Figure 4A, where the DOPC bilayer is 4 nm above the mica and the LH2 complexes are 6.5 nm above, in good agreement with the expected values [31]. A higher resolution image, shown in Figure 4C, shows a cluster of LH2 complexes in the DOPC bilayer with two height levels of LH2 apparent, as found in native membranes [16]. Alternatively, these height levels may represent an parallel/antiparallel orientations, as found in 2D crystals [5,46,47]. Because we cannot control orientations of complexes in proteoliposomes, it is likely that the LH2 complexes have inserted in both orientations, but the exact ratio cannot be determined from our data. The measured diameters of the LH2 complexes are approximately 6–7 nm. The AFM topographic image of an unbroken proteoliposome vesicle (Figure 4D) shows arrays of LH2 complexes arranged in a rectangular pattern, as previously reported for 2D crystals of LH2 [46]. Figure 4E,F shows a topograph of RC-LH1 only proteoliposomes, which reveals a lipid bilayer that contains dense arrays of RC-LH1 9-11 nm above the mica substrate (Figure 4F) in good agreement with the expected height of the complex [48]. As with LH2, we observe multiple heights for the RC-LH1 complexes, which could be a result of different orientations or packing of a curved complex on a flat surface for AFM imaging. We could not control the orientations of RC-LH1, so insertion in both parallel and anti-parallel orientations are possible. We note a lack of lipid-only membrane regions in our RC-LH1 proteoliposomes, which may indicate that only protein-rich regions adhered to the mica substrate for imaging or that all of the lipids in the reconstitution mixtures have been sequestered within and between the RC-LH1 complexes. Figure 4G,H shows the AFM images of a proteoliposomes containing a 1:1 ratio of LH2 and RC-LH1. The area marked with the rectangle in Figure 4G is shown at a higher resolution in Figure 4H. The LH2 complexes are indicated by red arrows, and the RC-LH1 complexes are indicated by blue arrows have diameters of approximately 8 and 12 nm, respectively. The images show that the RC-LH1 complexes, which are present in both the monomeric and dimeric forms, are closely associated with arrays of tightly packed LH2 complexes. This arrangement closely resembles that seen previously in chromatophores from high light-grown cells [16].

### Spectral analysis of fluorescence emission from LH2/RC-LH1 proteoliposomes

**Figure 5:**
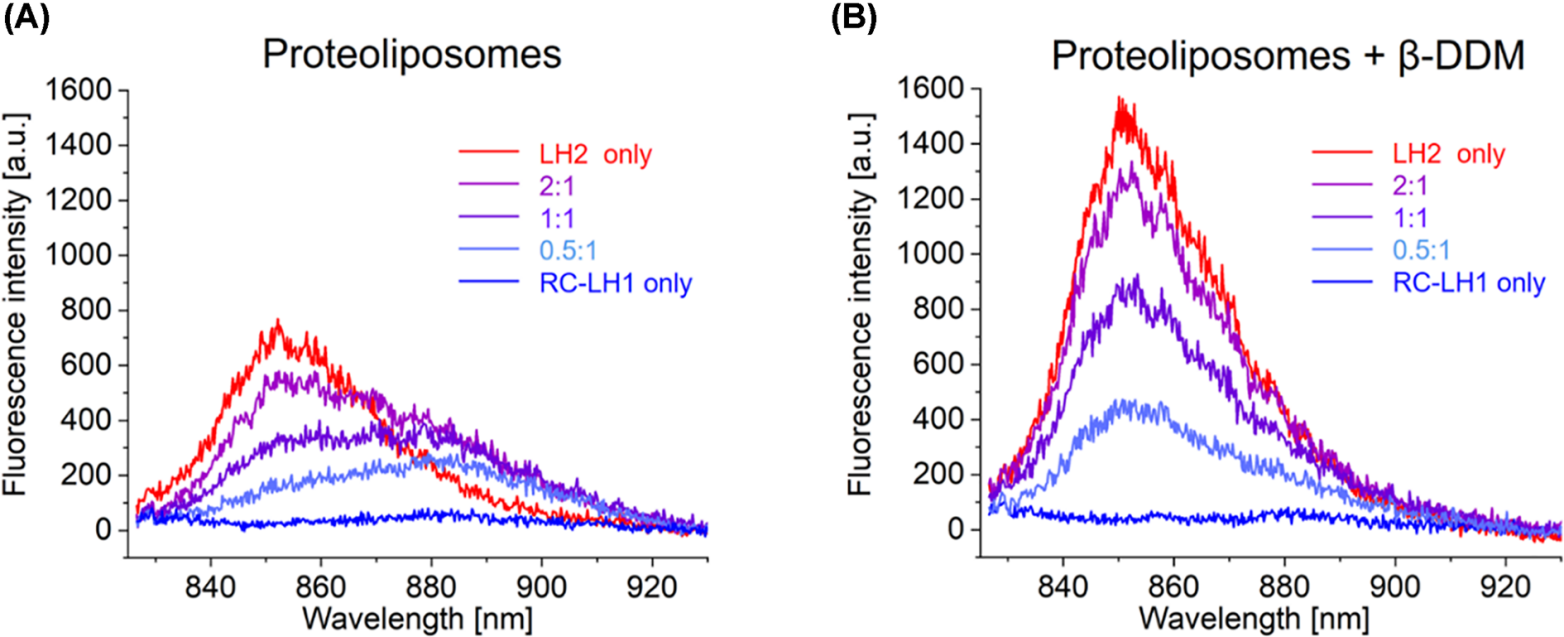
Fluorescence emission spectra of intact and detergent treated proteoliposome samples Each spectrum is an average of five measurements. (**A**) Fluorescence emission spectra of the five proteoliposome samples, with excitation of LH2 carotenoids at 485 nm (**B**) Fluorescence emission spectra of the proteoliposomes in (**A**) following addition of 2% β-DDM.

To monitor energy transfer from LH2 to RC-LH1, we collected steady-state fluorescence emission spectra of the proteoliposomes. The presence of carotenoids in LH2 but not in RC-LH1 complexes allows preferential excitation of LH2 at 485 nm, which gives a strong emission band at 855 nm (Figure 5A,B, red traces) because of efficient energy transfer from the carotenoid to the B850 BChls of LH2. As with the absorption spectra, the fluorescence emission spectra were similar for LH2 complexes in liposomes and detergent, indicating that reconstitution retains the structural and optical properties of the LH2 complexes. Owing to the lack of carotenoids in the RC-LH1 complexes, 485 nm excitation elicits only weak fluorescence emission at 883 nm from the LH1 B875 BChls in samples that do not contain LH2 complexes (Figure 5A,B, blue traces). Thus, observable emission from LH1 in mixed LH2/RC-LH1 proteoliposomes predominantly arises from transfer of excitation energy from adjacent LH2 complexes, and the expected signature of this energy transfer will be an increased LH1 emission at 883 nm with concomitantly lowered LH2 emission at 855 nm.

The fluorescence emission spectra for mixed LH2:RC-LH1 proteoliposome samples (2:1, 1:1, 0.5:1) in Figure 5A all show this signature, with LH1 emission at 883 nm increasingly apparent as the amount of LH2 increases, giving more possibilities for energy to migrate from LH2 to RC-LH1 complexes. Comparison of these mixed samples with the emission amplitude at 855 nm from LH2-only proteoliposomes shows that reconstitution allows some of the absorbed energy to transfer to RC-LH1. As the LH2:RC-LH1 ratio is increased from 1:1 to 2:1 emission from RC-LH1 rises only slightly, whereas LH2 emission increases by approximately 70%. This suggests that a significant proportion of the LH2 complexes within the 2:1 proteoliposomes are uncoupled from RC-LH1 complexes.

To provide a control, and further evidence that RC-LH1 complexes were receiving excitation energy from LH2, each proteoliposome sample was treated with 2% *n*-Dodecyl-β-D-maltopyranoside (β-DDM). This detergent releases the complexes from the membrane environment and from associations with each other, dispersing them in detergent micelles. As expected, following solubilisation all mixed micelles showed increased emission from the newly-uncoupled LH2 and minimal emission from RC-LH1 (Figure 5B). We note that the LH2 emission in proteoliposomes is blue-shifted by 2 nm upon β-DDM solubilisation, which correlates with a blue shift in the absorption maximum from 850 to 848 nm. These shifts are in keeping with observations by Pflock et al. who reconstituted LH2 complexes into proteoliposomes [45] and they reflect subtle changes to the environment of the LH2 BChls when transferred from a lipid to a detergent environment. Comparison of Figure 5A,B shows the increased emission intensity of donor LH2 complexes, which are now uncoupled from acceptor RC-LH1 complexes. As a result, the emission from these acceptors, which are unable to absorb 485 nm excitation energy, declines to undetectable levels.

### Fluorescence lifetime decay of LH2 complexes in proteoliposomes

**Figure 6:**
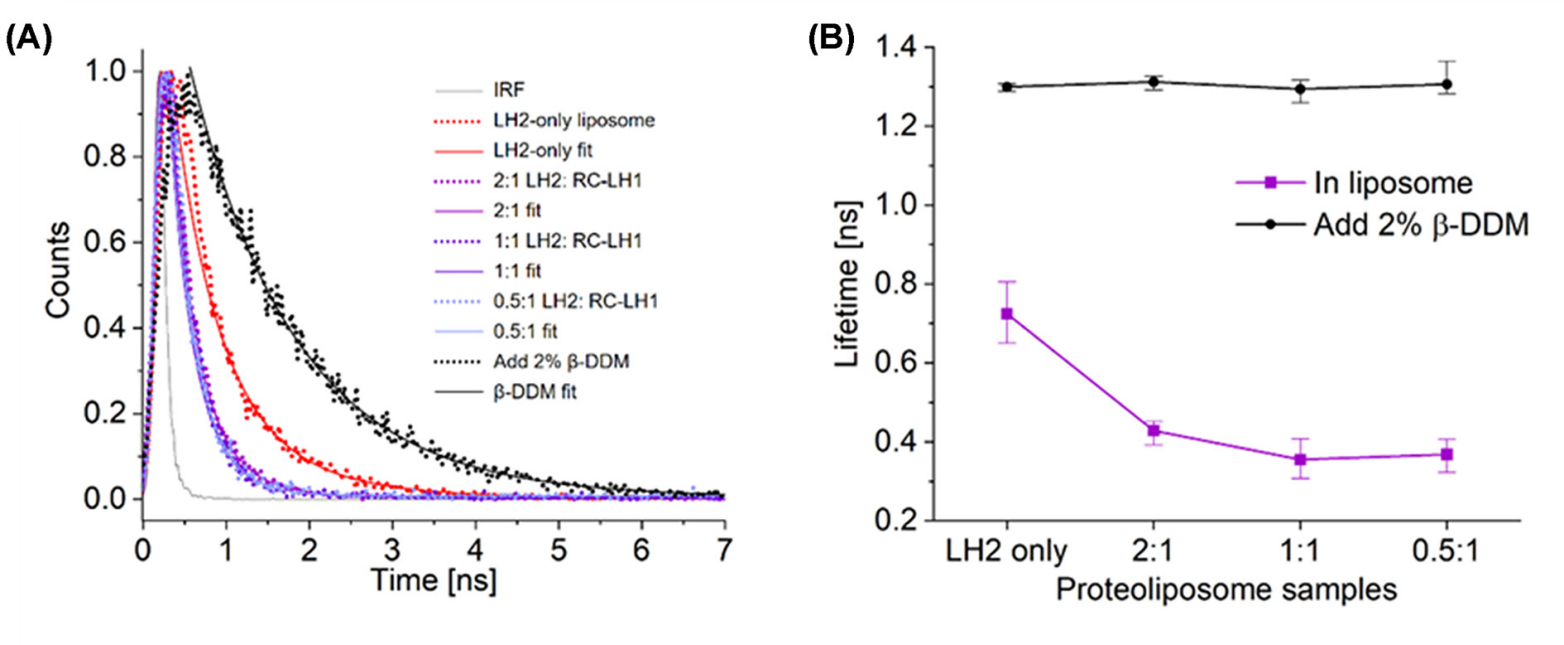
Fluorescence lifetimes of LH2 complexes in proteoliposomes and solubilised in β-DDM (**A**) Fluorescence lifetime decay curves of the LH2 complexes (dots) and their fitting results (solid lines); the instrument response function (IRF) was approximately 0.13 ns. (**B**) Fluorescence lifetimes of LH2 in proteoliposomes compared with the LH2 lifetime when the proteoliposomes were solubilised by 2% β-DDM.

To obtain more insight into energy transfer in the reconstituted photosystems, the lifetimes of fluorescence emission at 857 ± 3 nm were recorded following excitation at 485 nm. Figure 6A shows representative LH2 lifetime decay curves in proteoliposomes (dots) and their fits (solid lines). The curve for LH2-only proteoliposomes after solubilisation in 2% β-DDM is also shown. In an attempt to maintain RCs in an open state, 250 µM sodium D-ascorbate and 1 mM Coenzyme Q_0_ (an analogue of the native ubiquinone-10 lacking the isoprene tail) was added to the proteoliposomes. However, this treatment showed no effect on the amplitude or lifetime of LH2 fluorescence. Timpmann et al [41] showed that open RC traps shorten the overall fluorescence lifetime in membranes from high- and low-light grown cells of *Rba. sphaeroides*, from approximately 0.2 ns (closed) to 0.07 ns (open RCs). The values of ∼0.43–0.35 ns for LH2 lifetimes in the mixed micelles indicate that the decay kinetics were measured on LH2/RC-LH1 proteoliposomes with RCs in the closed state.

Figure 6B and Table 2 show the average fluorescence lifetimes of the LH2 complexes from the LH2-only and mixed LH2/RC-LH1 proteoliposomes and the same samples following solubilisation with 2% β-DDM. In all samples solubilised in 2% β-DDM, the fluorescence lifetimes of the LH2 complexes were around 1.3 ns, consistent with the unquenched state of the complex in its dispersed, monomeric state [49,50]. When only LH2 complexes are incorporated into proteoliposomes the complexes pack closely together, creating the conditions for LH2-LH2 energy transfer and the lifetime of emission from LH2 complexes decreases from 1.3 ± 0.01 to 0.72 ± 0.08 ns, similar to previous reports of around 0.7 ns [51,52]. The incorporation of RC-LH1 lowers the LH2 lifetime further, with values of 0.43 ± 0.04, 0.35 ± 0.05 and 0.37 ± 0.05 ns for proteoliposomes with a 2:1, 1:1 and 0.5:1 LH2:RC-LH1 ratio, respectively.

**Table 2:** Fluorescence lifetimes of LH2 complexes in proteoliposomes and when the proteoliposomes were solubilised by 2% β-DDM

Sample	Complexes present				
	LH2-only	RC-LH1 only	2:1 LH2:RC-LH1	1:1 LH2:RC-LH1	0.5:1 LH2:RC-LH1
Proteoliposomes (ns)	0.72 ± 0.08	n/a	0.43 ± 0.04	0.35 ± 0.05	0.37 ± 0.05
Solubilised proteoliposome (ns)	1.30 ± 0.01	n/a	1.31 ± 0.02	1.29 ± 0.04	1.31 ± 0.06
LH2→RC-LH1 energy transfer efficiency	n/a	n/a	40 ± 14%	51 ± 18%	49 ± 17%

By comparing the lifetimes for LH2-only and mixed LH2/RC-LH1 proteoliposomes, we see that addition of RC-LH1 energy acceptors always shortens the LH2 lifetime, so we use the ratio of mixed proteoliposome to LH2-only lifetime as an indicator of the extent of LH2 to RC-LH1 energy transfer. As already mentioned, the directionality of transfer was established by specifically exciting carotenoids, which are present only in LH2 donor complexes.

The extent of energy transfer from LH2 to RC-LH1 in mixed LH2/RC-LH1 proteoliposomes was calculated using the equation:

$$E=1-\frac{\tau_{DA}}{\tau_{D}}$$

where *τ_DA_* and *τ_D_* are the donor lifetime with and without the presence of the acceptor, respectively. In reconstituted proteoliposomes, excited LH2 complexes act as energy donors, delivering excitation energy to neighbouring RC-LH1 acceptors. As listed in Table 2, the LH2 emission lifetime of 0.72 ± 0.08 ns in LH2-only proteoliposomes decreases in the presence of RC-LH1 acceptors, and the efficiencies of energy transfer were 40 ± 14%, 51 ± 18% and 49 ± 17% for proteoliposomes with a 2:1, 1:1 and 0.5:1 LH2:RC-LH1 ratio, respectively. We note that within a given population of mixed LH2/RC-LH1 proteoliposomes there will be heterogeneities in LH2-LH2 and LH2-RC-LH1 contacts, and in the aggregation states for these complexes. There could also be LH2-only regions uncoupled from RC-LH1 acceptors. However, fitting of lifetimes to our data cannot deconvolute these effects, so the values obtained represent an average lifetime for the entire system rather than being representative of individual complexes.

## Discussion

In wild-type *Rba. sphaeroides*, light harvesting, energy transfer and trapping occur via two types of membrane protein complexes, LH2 and RC-LH1, the proportions of which vary according to the incident light levels [16]. This natural variation in the LH2 to RC-LH1 ratio provides some insight into how membrane architecture influences energy migration through the antenna network [41], and here we reconstituted LH2 and RC-LH1 complexes into liposomes, providing a minimal and controlled system for the functional analyses for light harvesting and energy transfer. In a previous study a fixed ratio of *Rhodopseudomonas palustris* LH2 and RC-LH1 complexes was reconstituted into phospholipids and evidence for excitation energy transfer from LH2 to RC-LH1 was obtained from steady-state and time-resolved fluorescence spectroscopy, with excitation of LH2 at 800 nm [50]. Finally, three LH2:RC-LH1 ratios were reconstituted into liposomes, using LH2 from *Rhodopseudomonas acidophila* and RC-LH1 from *Blastochloris viridis* [42] and excitation energy transfer was observed, on the basis of fluorescence emission spectroscopy. In our work, several LH2:RC-LH1 reconstitution ratios were examined, using a LH2/RC-LH1 system based on *Rba. sphaeroides*. The genetically engineered removal of carotenoids from RC-LH1 complexes enables selective excitation of LH2, and fluorescence emission and lifetime microscopy were used to demonstrate efficient energy transfer from LH2 to RC-LH1.

AFM images of the proteoliposomes show that clusters of LH2 self-assemble within the bilayer environment, separating into protein-rich and protein-free (empty) regions (Figure 4A-D), as also seen in another study [53]. In the proteoliposomes containing only RC-LH1 complexes, the membranes were packed with complexes and almost no lipid-only regions were imaged by AFM (Figure 4E,F). For proteoliposomes containing both LH2 and RC-LH1, AFM images show evidence that both cluster into the protein dense clusters (Figure 4G,H) with intermixing of LH2 and RC-LH1 complexes. Although we lack the ability to control the orientation of the complexes in the proteoliposomes, so they are likely to be inserted with a distribution of parallel and anti-parallel orientations, the overall arrangement seen in topographs of proteoliposomes (Figure 4) resembles that observed in natural chromatophore membranes imaged by AFM [16]. A recent study examined energy transfer between LH2/LH3 heterodimers from *Phaeospirillum molischianum* incorporated into lipid nanodiscs [54]. Cryogenic electron microscopy (cryo-EM) of nanodiscs showed there were mixed parallel and anti-parallel orientations; for smaller nanodiscs where the intercomplex distances are similar to those in native membranes, there were very similar nearest-neighbour BChl-BChl distances of 24.8 and 25.3 Å, respectively, and the same time constant of 5.7 ps for energy transfer between complexes. For larger nanodiscs with looser packing the numbers were 31.4 Å, 14.7 ps (parallel) and 28.5 Å, 9.8 ps (anti-parallel). In the case of parallel and anti-parallel orientations of adjacent LH2 and RC-LH1 complexes, we docked the cryo-EM structures [9,10], and found nearest-neighbour BChl-BChl distances were 24.7 and 26.6 Å, respectively (not shown), similar to those in LH2/LH3 pairs [54]. In summary, it is likely that mixed orientations of LH2 and RC-LH1 complexes still allow acceptable rates of energy migration among the complexes in our proteoliposome preparations.

Examination of the absorption spectra reveals that alterations are minimal upon reconstitution into liposomes, with a small red-shift being the most notable feature, as previously observed in reconstitutions of LH2s from other species [45]. We note that several factors may contribute to this red-shift including restoring protein–lipid interactions that are lost during detergent solubilisation, protein–protein interactions from contact with neighbouring LH complexes or, most likely, a combination of these. For example, a comparison of LH2 absorption for solubilised complexes, native membranes and 2-D crystals showed that absorption of native membranes or the 2-D arrays was red-shifted with respect to solubilised monomers; LH2 absorption of the crystals was slightly more red-shifted than for the membranes [55].

With the preferential excitation of the LH2 carotenoid, strong and quantifiable emission from LH1 is only apparent for mixed proteoliposomes and is almost absent from RC-LH1 only samples. This clearly shows that LH2 and RC-LH1 have packed together with their respective B850 and B875 rings within a few nanometres of one another, as expected from the clustering observed by AFM. Similarly, the inter-LH2 distances appear to be conducive to energy transfer as evidenced by the quenching of steady state fluorescence in LH2-only proteoliposomes, and the enhancement of quenching when trapping RC-LH1 complexes are introduced. Together, these results suggest that the reconstituted LH complex arrays in the proteoliposomes resemble those found in chromatophore membranes.

With clear evidence for the assembly of LH complex arrays and energy transfer within them, we sought to further characterise energy transfer by fluorescence lifetime microscopy. Our 1.3 ns lifetime for LH2 in detergent is comparable to the 0.93 ns lifetime measured by Pflock et al [45]. Upon reconstitution of LH2 into proteoliposomes these authors observed bi-exponential fluorescence decay curves with time constants of *τ*_1_ = 0.6-0.72 ns and *τ*_2_ = 0.07 ns, depending on the lipid:protein ratio. The authors concluded that the emergence of a fast 0.07 ns decay component and acceleration of the slow 0.6-0.72 ns component relative to the 0.93 ns lifetime in detergent arises from clustering of the LH2 complexes following reconstitution, promoting efficient energy transfer between complexes. Our lifetime of 0.72 ± 0.08 ns is comparable to these values and although we cannot measure a fast 0.07 ns component due to the 0.13 ns IRF of our instrument, we conclude that our fluorescence lifetime measurements also provide evidence for energy transfer between LH2 complexes in our system. Upon the addition of RC-LH1 to the proteoliposomes we observed a further reduction of lifetime that indicated effective energy transfer from LH2 B850 to B875 of LH1, where it is subsequently quenched by transfer to the RC, as supported by the AFM and steady state florescence measurements.

The LH2 to RC-LH1 stoichiometries used in this study have some effect on the extent of energy transfer. The 0.72 ± 0.08 ns fluorescence lifetime in LH2-only membranes decreases to 0.43 ± 0.04 ns with a ratio of 2:1 LH2 to RC-LH1, corresponding to 40 ± 14% energy transfer efficiency. When the ratio is lowered to 1:1 the lifetime shortens further to 0.35 ± 0.05 ns, but no further improvements are seen with a 0.5:1 LH2 to RC-LH1 ratio. This observation correlates with the intensity of steady state fluorescence where the LH1 emission is similar at all LH2 ratios, but the LH2 emission increases approximately linearly with the LH2 concentration. This trend can be explained by the arrangement of complexes within the liposome. At low LH2 to RC-LH1 ratios of 1:1 or less the LH2 only domains are small and most LH2 complexes are connected to RC-LH1 complexes for energy transfer. At higher LH2:RC-LH1 ratios the size of the LH2 domains grows, and some of the additional LH2 complexes are apparently uncoupled from RC-LH1 and cannot contribute to productive light harvesting and energy trapping.

Two-dimensional electronic spectroscopy has been used to follow all energy transfer processes in living cells of *Rba. sphaeroides*, including the wild-type, LH2-only and LH1-only strains [20]. The fluorescence lifetime for LH2-only membranes was found to be 0.25–0.3 ns, a smaller value than found for LH2-only proteoliposomes. For the wild-type, which in this case had a ratio of approximately 1.8 LH2:LH1, 83% of excitations were trapped by the RCs present. In these whole cell samples, the RCs were maintained in an open (reduced) state, available for efficient energy trapping. Timpmann et al. [41] examined a range of *Rba. sphaeroides* membranes, including LH2-only, RC-LH1 only and native membranes with contrasting LH2:RC-LH1 ratios. Fluorescence lifetimes varied from ∼0.49 ns for LH2-only membranes, to ∼0.25 ns for wild-type LH2/RC-LH1 membranes. The overall lifetime increased as the proportion of LH2 increased, consistent with the data on proteoliposomes containing 2:1 and 1:1 LH2:RC-LH1 ratios. These differences in lifetimes between our system and natural chromatophores could arise from a different lipid composition in the artificial membrane or differences in packing of the complexes, for instance by reconstituting RC-LH1 and LH2 complexes in non-uniform orientations. This reconstitution approach provides new possibilities for the creation of mix-and-match photosynthetic systems, for instance studying the possibility of energy transfer between photosynthetic complexes from different types of photosynthetic organisms, or between natural and artificial proteins. Future applications of our artificial system will enable the design and optimisation of systems that integrate native, non-native, engineered and *de novo* designed light harvesting complexes into functional photosynthetic systems.

## Materials and methods

### Protein purification

Wild type LH2 and Δ*crtB* RC-LH1 proteins were purified as described previously [55,56]. Briefly, cells were grown in 1.5 L M22+ medium [57] under semi aerobic conditions (in 2 L conical flasks shaken at 180 RPM at 34°C in darkness) for 72 h. Cells were harvested at 4,000 × ***g*** and broken via two passes through a French pressure cell (AmInCo, USA) at 18,000 psi, then unbroken cells and insoluble debris were removed by centrifugation at 25,000 × ***g*** for 15 min at 4°C. The supernatant was loaded onto a 40/15% w/w sucrose gradient and centrifuged at 100,000 × *g* for 10 h at 4°C in order to isolate the intracytoplasmic membranes (ICM). After harvesting, the ICMs were solubilised by addition of 3% (w/v) β-DDM for RC-LH1, or in 4% *N*,*N*-dimethyldodecylamine-*N*-oxide (LDAO) for LH2, stirring in the dark at 4°C for 45 min. The solubilized membrane solution was diluted at least three-fold in working buffer and centrifuged for 1 hour in a Beckman Ti 70.1 rotor at 48,000 rpm (160,000 × ***g***) at 4°C to remove unsolubilized material. The supernatant was further purified by using ion-exchange chromatography and concentrated using Amicon 100,000 MWCO spin filters (Millipore) in 10 mM HEPES pH 7.8, 50 mM NaCl, 0.03% (w/v) β-DDM buffer.

### Sample preparation

Liposomes were made by an extrusion method using DOPC lipid, by following the ‘Liposome Preparation Protocol’ provided by Avanti polar lipids (https://avantilipids.com/tech-support/liposome-preparation/). Specifically, DOPC was solubilised in chloroform at 10 mg/mL concentration. Approximately 150 μL of the solvent was evaporated to form DOPC lipid films, which were hydrated in 1 ml of buffer (20 mM MOPS, 20 mM NaCl, pH 7.8) and agitated by vortexing to produce a suspension of large, multilamellar vesicles (LMV). The stable and hydrated LMVs were extruded through a polycarbonate filter with 200 nm pores to form mono-layer vesicles of defined size. β-DDM was then added to the liposome solution at a final concentration of 0.03% w/v and incubated for 30 min before addition of purified light-harvesting complexes at a 500:1 mol/mol lipid:protein ratio and incubation for 1 h in the dark at 4°C. To remove β-DDM 10 mg/mL nonpolar polystyrene Bio-Beads (BIO-RAD, Bio-Beads SM-2 Adsorbents) were added to the solution and mixed gently (Stuart, SRT6) overnight at 4°C in the dark. Aggregated complexes that were not associated with liposomes were removed by sucrose density gradient centrifugation. Sucrose solutions were prepared in buffer (20 mM MOPS, 20 mM NaCl, pH 7.8) and the gradient formed with steps at 10%, 20%, 30%, 40% and 50% (w/w). Proteoliposomes were loaded onto the 10% sucrose layer and the gradients were centrifuged at 154,000 × ***g*** for 15 h at 4°C in an SW41Ti swinging bucket rotor (Beckman). Proteoliposome samples were carefully collected from the pigmented bands around the 25–30% interface using a peristaltic pump. The sizes of the reconstituted proteoliposomes were monitored by Dynamic Light Scattering (DLS).

### Room temperature absorbance spectra

Room-temperature absorbance spectra were recorded on a Cary 60 UV-Vis spectrophotometer (Agilent) at wavelengths between 250 and 1000 nm in an ultraviolet (UV) cuvette with a 1 cm path length. Baselines were corrected in the same range. Dilutions were made using the appropriate buffer or growth medium.

### Determination of extinction coefficients

RC-LH1 or LH2 was concentrated to a maximum OD of 50-100 (at 870 and 850 nm, respectively) using 100,000 MWCO centrifugal filters (Merck, U.S.A). A volume of 10 µL was added to 990 µl buffer (20 mM Tris pH 8, 200 mM NaCl, 0.03% β-DDM) or methanol and mixed gently by inversion followed by centrifugation at 13,000 RPM for 2 min in a benchtop microcentrifuge. UV/Vis/NIR spectra were immediately collected between 250 and 1000 nm. Five samples were prepared sequentially in both buffer and methanol and the entire procedure was carried out in the dark to minimise degradation of the BChl *a*.

To calculate the extinction coefficient, the absorbance at 771 nm for the methanol samples was averaged and the BChl *a* concentration was determined using an extinction coefficient of 54.8 mM^−1^ cm^−1^ [52,58]. To determine the complex concentration in each sample the BChl *a* concentration was divided by the number of BChl *a* molecules present per complex (32 for monomeric RC-LH1 or 27 for LH2). Extinction coefficients for the intact complexes were determined by averaging the maximal absorbance for the samples in buffer (875 nm for RC-LH1 or 850 nm for LH2) according to (eqn 1) where *A* is the average absorbance in buffer, and *C* is the calculated complex concentration from the methanol extractions:

(1) $$\varepsilon =\frac{A}{C}$$

The resulting extinction coefficients were 3000 ± 20 mM^−1^ cm^−1^ at 875 nm for RC-LH1 and 2910 ± 50 mM^−1^ cm^−1^ at 850 nm for LH2.

### Calculation of protein concentration in proteoliposomes

Room-temperature absorbance spectra were processed by scatter correcting and deconvoluting the contributions of RC-LH1 and LH2 as described previously [6]. Briefly, reference spectra, and a scatter curve calculated using ʎ^−2.6^ were scaled to give a best fit to the experimental data in Microsoft Excel. Concentrations of RC-LH1 and LH2 were determined from their calculated components using the calculated extinction coefficients.

### Characterisation of proteoliposomes by atomic force microscopy

The AFM topographs were collected on a Multimode 8 instrument equipped with a 15 μm scanner (E-scanner) coupled to a NanoScope V controller (Bruker). NanoScope software (v9.2, Bruker) was used for data collection and Gwyddion (v2.52, open-source software covered by GNU general public license, www.gwyddion.net) and OriginPro (v8.5.1, OriginLab Corp.) software packages were used for data processing and analysis. Proteoliposome samples were incubated on mica discs for 1 h at 4°C in adsorption buffer (20 mM MOPS pH 7.8, 20 mM NaCl and 5 mM MgCl_2_), then imaged in imaging buffer (20 mM MOPS pH 7.8 and 20 mM NaCl). AFM images were recorded in peak-force tapping mode at a peak-force frequency of 2 kHz, using SNL-10 probes (56 kHz, k∼0.24 Nm^−1^) (Bruker Nano). The peak-force amplitude was 10 nm and images were taken using either 256 × 256 or 512 × 512 pixel arrays. The peak-force set point varied between 50 and 1000 pN and the scan rate was between 0.5 and 1.0 Hz.

### Fluorescence life-time microscopy (FLIM)

Fluorescence emission spectra and lifetimes were measured on a home-built time-resolved fluorescence microscope equipped with a 485 nm picosecond diode laser (PicoQuant, PDL 828) for spectral and lifetime measurements. The excitation light is focused by a 100 × objective (PlaneFluorite, NA = 1.4, oil immersion, Olympus) and the fluorescence emission is collected from the same focal spot on the sample. The collected light is then filtered by a 495 nm dichroic beam-splitter to remove the background excitation light. A spectrometer (Acton SP2558, Princeton Instruments) was equipped for wavelength selection. An electron-multiplying charge-coupled device (EMCCD) detector (ProEM 512, Princeton Instruments) was equipped for spectral recording and a hybrid detector (HPM-100-50, Becker & Hickl) was equipped for photon counting. The modulation of the laser was synchronized with a time-correlated single-photon counting (TCSPC) module (SPC-150, Becker & Hickl) for the lifetime decay measurement. Samples were excited by the 485 nm pulsed laser at 1 MHz repetition rate and fluence of ∼2 × 10^14^ photons pulse^−1^ cm^−2^. TCPSC was applied for triggering the laser and counting the photon arrival time. TCPSC is a well-established and a common technique for fluorescence lifetime measurements. It detects single photons and measures their arrival times in respect to the light source. For the measurements in this work, the entrance slit of the spectrometer was closed to 100 µm. A grating with 150 lines/mm was used to select the wavelength. An 857/30 nm bandpass filter for LH2 and a 900/32 nm for RC-LH1, together with a secondary exit slit on the spectrometer, were used to narrow the recording wavelength range to 3 nm.

The fluorescence decay curves were analysed in OriginPro and TRI2 (open source), with fitting using the multi-exponential decay function:

$$I\left(t\right)=A_{1}\exp\left(\frac{-t}{\tau_{1}}\right)+A_{2}\exp\left(\frac{-t}{\tau_{2}}\right)+B$$

Where *τ* is the fluorescence lifetime, *A* is the fractional amplitude contribution of the decay component, and *B* is the background. The quality of the fit was judged on the basis of the reduced *χ*^2^ statistic:

$$\chi_{\text{red}}^{2}=\frac{\sum_{\text{k}=1}^{\text{n}}([\text{I}({\text{t}}_{\text{k}})-{\text{I}}_{\text{c}}({\text{t}}_{\text{k}}){]}^{2}/\text{I}({\text{t}}_{\text{k}}))}{\text{n}-\text{p}}=\frac{\chi^{2}}{\text{n}-\text{p}}$$

where *t_k_* is the time point *k*, *I(t_k_)* is the data at the time point *k, I_c_(t_k_)* is the fit at the time point *k, n* is the number of the data points and *p* is the number of the variable fit parameters *(n - p =* degrees of freedom*).*

Using a mirror to replace the sample, the time delay of the laser from the pulse starting point to the instrument responding point was measured. Such time delay was defined as the instrument response function (IRF), which was approximately 0.13 ns on the home-built fluorescence microscope. The IRF was taken into account when the fitting was performed for the decay curves.

## Supplementary Material

Supplementary Figures S1-S3 and Table S1

## Data Availability

The processed data required for interpretation of our results are provided within the manuscript and supporting information. Raw data will be made available upon request.

## Open Access

Open access for this article was enabled by the participation of University of East Anglia in an all-inclusive *Read & Publish* agreement with Portland Press and the Biochemical Society under a transformative agreement with JISC.

## Abbreviations

β-DDM: *n*-dodecyl-β-D-maltopyranoside
cryo-EM: cryogenic electron microscopy
EMCCD: electron-multiplying charge-coupled device
ICM: intracytoplasmic membrane
IRF: instrument response function
LH2: light-harvesting 2 complex
RC: reaction centre
RC-LH1: RC-light-harvesting 1 complex
TCSPC: time-correlated single-photon counting
