# Supplementary material for: Excitation energy transfer in proteoliposomes reconstituted with LH2 and RC-LH1 complexes from Rhodobacter sphaeroides
Source: Biosci Rep. 2024 Feb 19;44(2):BSR20231302. doi: 10.1042/BSR20231302 (PMC10876425; doi:10.1042/BSR20231302)
Supplement: Supplementary Figures S1-S3 and Table S1 [file BSR-2023-1302_supp.pdf]

6. Supplementary Information

6.1 Size distribution of LH2 proteoliposomes

DLS measurements were taken for the LH2-only proteoliposomes, immediately following reconstitution and after separation from free and aggregated complexes by sucrose gradient centrifugation. The size distribution curves from the two measurements are shown in Figure S1. Only one peak is seen for both distribution curves, which means the LH2 proteoliposomes were relatively uniform in size as a monodisperse sample both before and after the sucrose gradient.

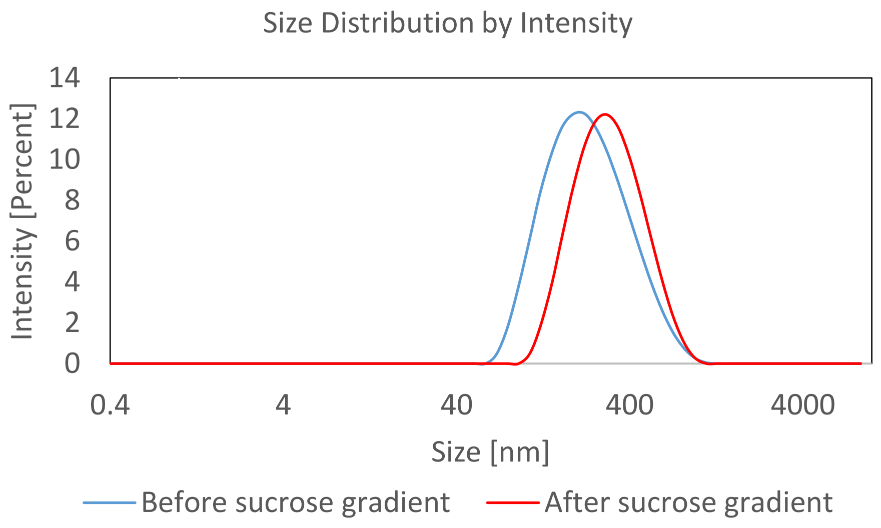

Figure S1. Size distribution of LH2-only proteoliposomes. The blue curve shows the size distribution of LH2 proteoliposomes following the reconstitution; the red curve shows the size distribution of LH2 proteoliposomes after the sucrose gradient.

Statistics of size distribution curves are listed in Table S1. The peak position corresponds to the size at the strongest scattering intensity; the average size stands for the intensity-based overall average size; the polydispersity index (PdI) indicates the width of the overall distribution; a monodisperse sample would have a low PdI, and a PdI greater than 0.7 indicates that the sample has a broad size distribution. The strongest scattering was from proteoliposomes with diameters of 257 nm and 328 nm before and after sucrose gradient separation, respectively. The average sizes of the proteoliposomes are 197 nm and 262 nm, close to the extrusion filter pore size of 200 nm. For both measurements, the PdI values are reasonably low, just below 0.2.

Sucrose gradient	Peak size (nm)	Average size (nm)	PdI
Before	257	197	0.199
After	328	262	0.199

Table S1. Size distribution of LH2-only proteoliposomes.

6.2 Arrangement of complexes in proteoliposomes

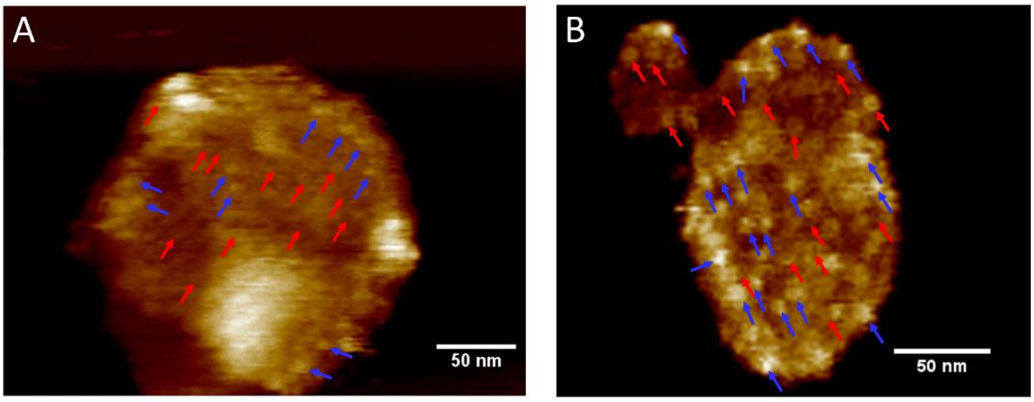

Figure S2. AFM topographs of proteoliposomes. (A) AFM image with LH2 to RC-LH1 ratio of 2:1. (B) AFM image with LH2 to RC-LH1 ratio of 0.5:1. Tentative positions of LH2 complexes are indicated by red arrows and the RC-LH1 complexes are indicated by blue arrows.

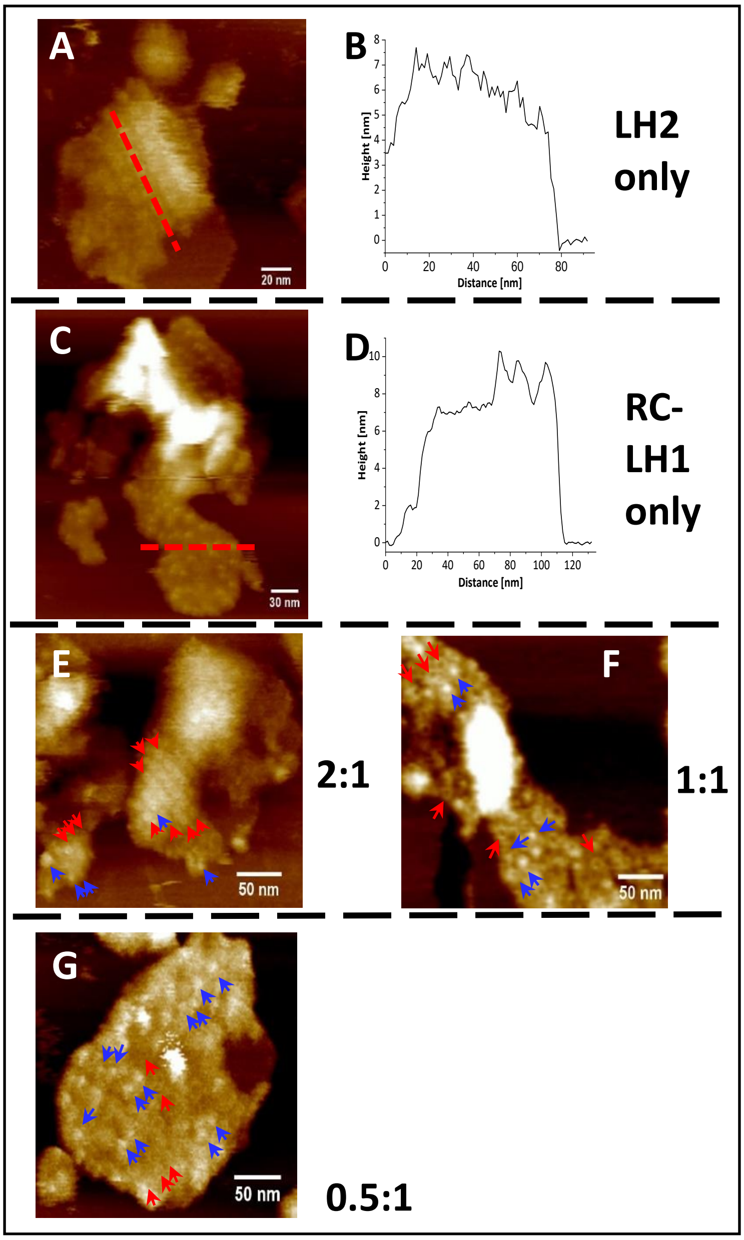

Figure S3. Additional AFM topographs of proteoliposomes. (A) LH2-only proteoliposome showing LH2 complexes (pale brown) sitting next to a region of empty DOPC bilayer (brown). (B) The corresponding height profile along the red dashed line in panel A, showing 5.5-7.5 nm height maxima corresponding to LH2. (C) A topograph of an RC-LH1 only proteoliposome. (D) The corresponding height profile along the red dashed line in panel C. (E-G) AFM topographs with LH2 to RC-LH1 ratios of 2:1, 1:1, and 0.5:1, respectively. Tentative positions of LH2 complexes are indicated by red arrows and the RC-LH1 complexes are indicated by blue arrows.
